# Historical and Current Adenosine Receptor Agonists in Preclinical and Clinical Development

**DOI:** 10.3389/fncel.2019.00124

**Published:** 2019-03-28

**Authors:** Kenneth A. Jacobson, Dilip K. Tosh, Shanu Jain, Zhan-Guo Gao

**Affiliations:** Molecular Recognition Section, Laboratory of Bioorganic Chemistry, National Institute of Diabetes and Digestive and Kidney Diseases, National Institutes of Health, Bethesda, MD, United States

**Keywords:** purinergic signaling, adenosine receptors, inflammation, pain, CNS

## Abstract

Adenosine receptors (ARs) function in the body’s response to conditions of pathology and stress associated with a functional imbalance, such as in the supply and demand of energy/oxygen/nutrients. Extracellular adenosine concentrations vary widely to raise or lower the basal activation of four subtypes of ARs. Endogenous adenosine can correct an energy imbalance during hypoxia and other stress, for example, by slowing the heart rate by A_1_AR activation or increasing the blood supply to heart muscle by the A_2A_AR. Moreover, exogenous AR agonists, antagonists, or allosteric modulators can be applied for therapeutic benefit, and medicinal chemists working toward that goal have reported thousands of such agents. Thus, numerous clinical trials have ensued, using promising agents to modulate adenosinergic signaling, most of which have not succeeded. Currently, short-acting, parenteral agonists, adenosine and Regadenoson, are the only AR agonists approved for human use. However, new concepts and compounds are currently being developed and applied toward preclinical and clinical evaluation, and initial results are encouraging. This review focuses on key compounds as AR agonists and positive allosteric modulators (PAMs) for disease treatment or diagnosis. AR agonists for treating inflammation, pain, cancer, non-alcoholic steatohepatitis, angina, sickle cell disease, ischemic conditions and diabetes have been under development. Multiple clinical trials with two A_3_AR agonists are ongoing.

## Introduction

Adenosine receptors (ARs) are G protein-coupled receptors (GPCRs) that sense an imbalance of demand and supply of energy/oxygen/nutrients. Extracellular adenosine concentrations rise in response to hypoxia and other stress, to act upon four subtypes of ARs (A_1_AR, A_2A_AR, A_2B_AR, and A_3_AR) (Fredholm et al., 2001; Chen et al., 2013). As shown with mice lacking all four AR subtypes, extracellular adenosine is mainly a sensor of tissue damage or danger, rather than a homeostatic regulator under baseline conditions (Xiao et al., 2019). Elevated adenosine can correct an energy imbalance during distress of an organ, for example by slowing the heart rate by A_1_AR activation or increasing the blood supply to heart muscle by the A_2A_AR (Borea et al., 2016). However, there are conditions in which chronic adenosine overproduction can be harmful, leading to increased inflammation, fibrosis, cytokine release, brain dopamine depletion, and kidney damage (Borea et al., 2017). Moreover, exogenous AR agonists, antagonists, or allosteric modulators can be applied for therapeutic benefit, and thousands of such agents have been reported by medicinal chemists working toward that goal (Jacobson and Gao, 2006; Kiesman et al., 2009). Clinically important effects of adenosine also include suppression of the immune response, glomerular filtration, seizures and pain (Fredholm et al., 2001; Cekic and Linden, 2016; Antonioli et al., 2018). Adenosine 5′-triphosphate (ATP) released from cells under stress conditions or damage is the source of much of the extracellular adenosine. There is typically a basal level of AR stimulation, especially for A_1_AR, A_2A_AR and A_3_AR at low nM concentrations, while A_2B_AR activation generally occurs at higher (μM) adenosine concentrations (Fredholm et al., 2001). Therefore, AR antagonists have distinct biological effects *in vivo*. Purinergic signaling is also to be considered in the larger context of ligand (ATP)-gated P2X receptors or G protein-coupled P2Y receptors that respond to extracellular purine and pyrimidine mono- and di-nucleotides (Burnstock and Boeynaems, 2014).

There are many approaches to small molecule therapeutics based on experimental modulators of ARs (Chen et al., 2013; Borea et al., 2018). Numerous subtype-selective agonists and antagonists have been introduced, both as pharmacological probes and as clinical candidate molecules, and representative compounds are described here. AR knockout (KO) mice are increasingly used to validate *in vivo* results with agonists and antagonists, which can actually have variable selectivity (Carlin et al., 2018). This review covers both directly acting AR modulators, i.e., agonists, and several positive allosteric modulators (PAMs). It describes compounds that have been in human trials, for both therapeutics and diagnostics, and some compounds for which clinical trials have only been contemplated. Other reviews cover the use of enzymatic or transport inhibitors to increase – such as inhibitors of adenosine kinase or adenosine deaminase that deplete adenosine levels, or uptake inhibitors – or to decrease, such as inhibitors of ectonucleotidases, the levels of endogenous nucleosides (Boison, 2013; Allard et al., 2017; Vuerich et al., 2019). These enzymes are often upregulated in inflammatory states (Boison, 2013; Haskó et al., 2018).

## AR Agonists for Clinical Development

**FIGURE 1:**
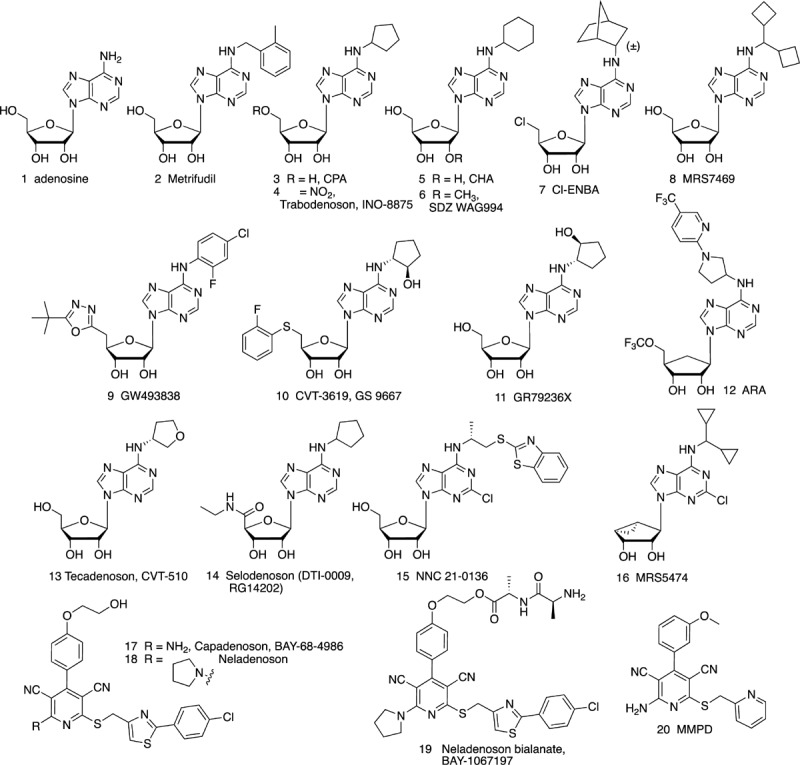
Adenosine **(1)**, a non-selective AR agonist, and its derivatives as A_1_AR-selective agonists, including nucleosides **(2**–**16)** and non-nucleosides **(17**–**20)**.

**FIGURE 2:**
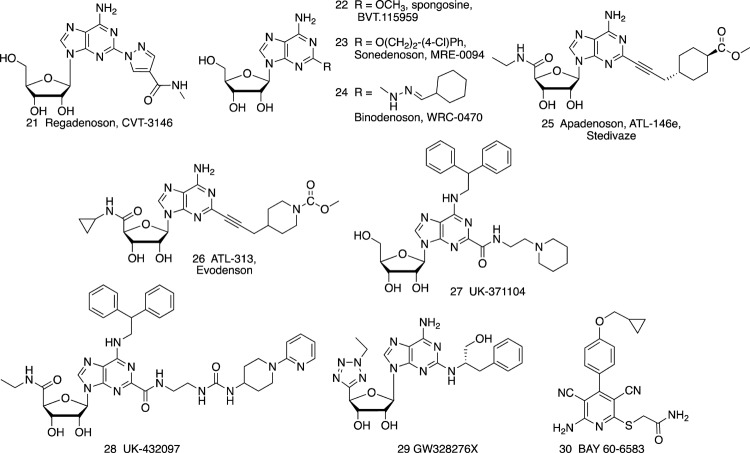
A_2A_AR- **(21**–**29)** and A_2B_AR- **(30)** selective agonists.

Agonists of the A_1_AR, A_2A_AR and A_3_AR have been the subject of preclinical and clinical evaluation (Figure 1, 2 and Tables 1, 2). A_2B_AR agonist development is the most limited among the ARs, and agonists for this AR subtype have not yet entered clinical trials. There is also much controversy about whether A_2B_AR agonists or antagonists would be more useful clinically (Eisenstein et al., 2015; Sun and Huang, 2016; Gao and Jacobson, 2017).

**Table 1:** Representative recent clinical trials of AR agonists and an A_1_AR PAM (data from ClinicalTrials.gov, accessed 12-28-2018).

Receptor	Condition	Compound	Years	Phase, NCT#	Reference
A_1_	Neuropathic pain	**1**	2014–2018	**2**, 00349921	Zylka, 2011
	SVT^a^	**1**	2011–2017	**-**, 01495481	Chrysostomou et al., 2013
	Perioperative pain	**1**	2006	**2**, 00298636	Jin and Mi, 2017
	Pediatric heart transpl.	**1**	2015–2018	**1**, 02462941	Flyer et al., 2017
	Takotsubo syndrome	**1**	2016–2018	**2**, 02867878	Galiuto et al., 2010
	Paroxysmal AF	**1**	2017	**2**, 03032965	Letsas et al., 2017
	Glaucoma	**4**	2015–2016	**3**, 02565173	Myers et al., 2016
	Neuropathic pain	**9**	2002–2003	**2**, 00376454	Elzein and Zablocki, 2008
	T2D	**10**	∼2009	**1**, **-**	Staehr et al., 2013
	AF	**13**	2008–2014	**2**, 00713401	Corino et al., 2015
	AF	**14**	2015–2018	**2**, 00040001	Elzein and Zablocki, 2008
	Stable angina	**17**	2007–2011	**2**, 00518921	Tendera et al., 2012
	AF	**17**	2008	**2**, 00568945	Kiesman et al., 2009
	Heart failure^b^	**19**	2014–2016	**2**, 02040233	Lam et al., 2018
	Heart failure^c^	**19**	2016–2018	**2**, 02580851	Voors et al., 2017
	Posthepetic neuralgia	**39**	2008–2012	**2**, 00809679	Miao et al., 2018
A_2A_	CAD (MPI)	**21**	2005–2009	**3**, 00208312	Zoghbi and Iskandrian, 2012
	Sickle cell anemia	**21**	2013–2018	**2**, 01788631	Field et al., 2014, 2017
	Lung transplant	**21**	2017-	**1**, 03072589	Sharma et al., 2016
	IHD (MRI)	**21**	2011–2012	**1**, 00881218	Lasley, 2018
	Pulmonary hypertens.	**21**	2014–2018	**-**, 02220634	Palani and Ananthasubramaniam, 2013
	Heart transplant (MRI)	**21**	2017-	**4**, 03102125	Palani and Ananthasubramaniam, 2013
	BBB defect	**21**	2015–2018	**1**, 02389738	Jackson et al., 2018
	Diabetic nerve pain	**22**	2007–2014	**2**, 00452777	Knezevic et al., 2015
	Diabetic foot ulcers	**23**	2006–2012	**2**, 00318214	Montesinos et al., 2015
	CAD	**24**	2009–2012	**3**, 00944294	Zoghbi and Iskandrian, 2012
	CAD (SPECT-MPI)	**25**	2009–2012	**3**, 00990327	Zoghbi and Iskandrian, 2012
	CAD (MPI)	**25**	2011–2012	**3**, 01313572	Zoghbi and Iskandrian, 2012
	COPD	**28**	2007–2013	**2**, 00430300	Mantell et al., 2010
	Healthy subjects (PK)	**29**	2012–2017	**1**, 01640990	Allen et al., 2013
A_2B_- A_3_		–	–		Sun and Huang, 2016
	Rheumatoid arthritis	**31**	2016-	**3**, 02467762	Fishman et al., 2012
	Plaque psoriasis	**31**	2017-	**3**, 03168256	David et al., 2016;
	Glaucoma	**31**	2009–2015	**2**, 01033422	Jacobson and Civan, 2016
	Dry eye disease	**31**	2010–2015	**3**, 01235234	Avni et al., 2010
	NASH	**32**	2016-	**2**, 02927314	Fishman et al., 2018
	HCC	**32**	2008–2015	**1/2**, 00790218	Stemmer et al., 2013
	HCC	**32**	2014-	**2**, 02128958	Stemmer et al., 2013
	Chronic HCV	**32**	2008–2015	**1/2**, 00790673	Bar-Yehuda et al., 2008

*^a^Comparison to dexmedetomidine. ^b^With preserved ejection fraction. ^c^With reduced ejection fraction. SVT, supraventricular tachycardia; AF, atrial fibrillation; CAD, coronary artery disease; MPI, myocardial perfusion imaging; MRI, magnetic resonance imaging; SPECT, single-photon emission computed tomography; BBB, blood brain barrier; IHD, ischemic heart disease; PK, pharmacokinetics; NASH, nonalcoholic steatohepatitis; HCC, hepatocellular carcinoma; HCV, hepatitis C virus.*

**Table 2:** AR Binding affinity of selected AR agonists described here (human, if not specified; p, pig; r, rat; m, mouse).^a,b^

Compound		pK_i_ A_1_AR	pK_i_ A_2A_AR	pK_i_ A_3_AR
1	Adenosine	7.0	6.5	6.5
2	Metrifudil	7.22 (r)	7.62 (r)	7.33, 7.46 (r)
3	CPA	8.64, 9.66 (m)	6.10, 6.09 (m)	7.37, 6.27 (m)
4	Trabodenoson	9.0	ND	ND
5	CHA	8.62	5.86	7.14
6	SDZ WAG994	7.64 (p), 7.12 (r)	4.64 (p), 5.24 (r)	ND
7	Cl-ENBA	9.29, 9.70 (m)	5.87	5.89, 5.62 (m)
8	MRS7469	8.67, 9.43 (m)	5.45	4.97, 6.05 (m)
10	GS 9667	7.92	<5	<6
11	GR79236X	8.51 (r)	5.89 (r)	ND
13	Tecadenoson	8.52	ND	ND
14	Selodenoson	8.22	ND	ND
15	NNC 21-0136	8.33 (r)	5.89 (r)	ND
16	MRS5474	7.30	5.40	6.33
17	Capadenoson	8.85	<5	<5
18^c^	Neladenoson	10.0	6.17	<5.52
20	MMPD	9.31, 9.68 (r)	7.15, 7.28 (r)	<5
21	Regadenoson	<5, 8.11 (m)	6.34, 7.11 (m)	<5, <5 (m)
22	BVT.115959	6.81	6.01	6.81
23	Sonedenson	<5	6.31	ND
24	Binodenoson	4.32	6.57	<4
25	Apadenson	7.11	9.30	7.35
26	Evodenoson	7.24	9.15	6.60
27^c^	UK-371104	6.99	7.70	<6
28	UK-432097	ND	8.40	ND
29	GW328276X	6.05	8.63	8.38
30	Bay 60-6583	6.41, 6.45 (m)	<5, <5 (m)	6.94, 6.87 (m)
31	Piclodenoson	7.29, 7.79 (m)	5.50, 5.54 (m)	8.74, 9.66 (m)
32	Namodenoson	6.66	5.27	8.85
33	LJ-529	6.71	6.65	9.42
34	CP-532,903	6.05 (m)	<5 (m)	8.05 (m)
35	CP-608,039	5.14	<4.3	8.24
36	MRS5698	<5	<5	8.52
37	MRS5980	<5	<5	9.15

*^a^References: Knutsen et al., 1999; Gao et al., 2003; Kiesman et al., 2009; Alnouri et al., 2015; Meibom et al., 2017; Baraldi et al., 2018; Jacobson et al., 2018; Tosh et al., 2019. ^b^A_2B_AR affinity (pKi; h, unless noted): **1**, 4.8; **18**, 7.10 (pEC_50_); **21**, <5; **23**, <5; **25**, <6; **26**, <6; **27**, 5.36 (pEC_50_); **29**, 8.30; **30**, 6.94, 7.00 (r), 6.87 (m); **31**, 4.96; **34**, <5 (m). ^c^pEC_50_ in cAMP assays. ND, not determined.*

Each AR can signal through multiple pathways, and different agonists may show a signaling preference, i.e., bias. For example, the A_2B_AR acts through G_s_, G_q_, or G_i_, depending on the tissue, receptor density and final measure of activity (Gao et al., 2018). The possibility of biased signaling of AR agonists or PAMs could provide a means to increase selectivity for a particular tissue or condition (Gao et al., 2011; Baltos et al., 2017; Gao et al., 2018; Vecchio et al., 2018).

One limitation of AR agonists for human therapeutics is that their target GPCRs might be subject to agonist-induced desensitization as shown for all four ARs in cell lines (Mundell and Kelly, 2011). However, in other cases, depending on multiple pharmacological factors, a prolonged agonist exposure did not lead to *in vivo* desensitization of the desired effect (Little et al., 2015), even with a full A_3_AR agonist. Two possible approaches to circumvent GPCR desensitization are the use of partial agonists in cases where the desired effect is maintained, such as in A_1_AR antiarrhythmic activity (Elzein and Zablocki, 2008; Voors et al., 2017), and the use of PAMs (Vincenzi et al., 2014). Partial agonists are also known to display tissue or organ selectivity, because of the differential receptor expression level (Van der Graaf et al., 1999). Thus, when spare receptors are present, i.e., overexpression or high level of endogenous expression, a partial agonist could be fully efficacious in tissues where there is a receptor reserve and activation of only a fraction of the receptors suffices.

Positron emission tomography (PET) for *in vivo* imaging of ARs has been extensively explored (van Waarde et al., 2018). PET imaging could potentially be essential in drug discovery and clinical development of AR modulators, to determine the target engagement and to assess the role of endogenous adenosine. Although most high affinity ligands designed for *in vivo* AR imaging by PET have been antagonists, some agonists have also been labeled with positron-emitting ^18^F, ^76^Br or ^11^C isotopes (Kiesewetter et al., 2009; Guo et al., 2018).

A_1_AR and A_2A_AR are examples of the growing list of GPCRs for which physically determined three-dimensional structures with a bound agonist are known (Lebon et al., 2011; Xu et al., 2011). This understanding facilitates the structure-based design of novel AR agonists (Tosh et al., 2019). Several structures determined by X-ray crystallography and cryo-electron microscopy contain a G-protein or G-protein fragment, which is more representative of the active state AR structures (Draper-Joyce et al., 2018; García-Nafría et al., 2018). NMR studies of assigned A_2A_AR Trp and Gly residues are informative about microswitches involved in the signal propagation from bound agonist toward the intracellular region for G protein binding (Eddy et al., 2018). There can be multiple active conformations of a given GPCR, and agonist-induced changes are nuanced with respect to both structure and signaling. Thus, direct biophysical methods for receptor characterization can impact agonist design.

The following examples of AR drugs and molecular probes are arranged according to their target receptor: A_1_ (**1** – **20**, **39**, **40**), A_2A_ (**21** – **29**), A_2B_ (**30**), A_3_ (**31** – **38**, **41**). It has also been suggested that coactivation of two AR subtypes might be beneficial, such as both A_1_AR and A_3_AR in cardioprotection (Jacobson et al., 2005).

### Early AR Agonists

#### Adenosine **1**

##### Arrhythmias

Many pathophysiological conditions including hypoxia and ischemia may cause arrhythmias. Adenosine is considered as an endogenous antiarrhythmic agent partly due to its endogenous anti-ischemic property (Kiesman et al., 2009; Szentmiklosi et al., 2015). As described earlier, infused adenosine (under the name of Adenocard, approved in 1989, Olsson, 2003) and its precursor ATP have long been used for the treatment of paroxysmal supraventricular tachycardia (PSVT) (Pelleg et al., 2012; Szentmiklosi et al., 2015). The antiarrhythmic action of adenosine has been suggested to occur via the A_1_AR in the sinoatrial and atrioventricular nodes, which leads to modification of AV nodal conduction (Kiesman et al., 2009). A_1_AR activation is known to induce opening of ATP-sensitive potassium channels (Fredholm et al., 2001; Jacobson and Gao, 2006). Adenosine infusion (under the name of Adenoscan, approved in 1995) is used for myocardial perfusion imaging (MPI), through its short-lived A_2A_AR activation, to dilate the coronary artery (Olsson, 2003). Many recent clinical trials in various cardiovascular and ischemic conditions have utilized adenosine as an approved drug being tested for new uses (Table 1). For example, a clinical trial of adenosine to reverse left ventricular impairment in Takotsubo syndrome (Galiuto et al., 2010) was initiated, but terminated in 2018.

##### Dermatological conditions

Adenosine applied topically has been used to promote hair growth and skin health (Abella, 2006; Faghihi et al., 2013). For the treatment of androgenetic alopecia, adenosine (0.75% solution) displayed efficacy similar to minoxidil but was preferred by patients because of the response speed and its quality. Applied in cosmetic preparations (0.1% cream or 1% dissolvable film) for 2 months, adenosine significantly improved skin smoothness and reduced facial wrinkles.

##### Epilepsy

Adenosine is considered an endogenous antiseizure agent and also attenuates epileptogenesis by an epigenetic mechanism, i.e., nuclear adenosine reduces DNA methylation (Boison, 2013). Elevated adenosine indirectly inhibits DNA methyltransferases through two stages of enzymatic product inhibition, beginning with *S*-adenosylhomocysteine hydrolase (SAH). In the epileptic brain there is an overexpression of adenosine kinase in astrocytes, which depletes adenosine both inside and outside the cell. Because peripheral adenosine itself is rapidly removed from circulation in seconds, drug delivery systems are needed to raise its brain concentration. Experimental use of an adenosine-releasing silk brain implant was found to be an efficacious form of adenosine augmentation therapy (AAT) for treating refractory epilepsy in a rat model of kindling epileptogenesis (Szybala et al., 2009). Adenosine was encapsulated in microspheres, which were embedded into nanofilm-coated silk fibroin scaffolds. The polymer was introduced surgically into the infrahippocampal cleft, and when monitored for 10 days, demonstrated a lack of convulsive seizures. This protective effect was antagonized by an A_1_AR-selective antagonist 8-cyclopentyl-1,3-dipropylxanthine (DPCPX).

#### AMP (Adenosine 5′-Monophosphate) and ATP (Adenosine 5′-Triphosphate)

##### Asthmatic drug testing

Ectonucleotidases readily cleave the naturally occurring 5′-phosphoesters of adenosine 5′-phosphates to produce adenosine *in situ*. Adenosine 5′-monophosphate (AMP) is used diagnostically in inhalation challenge testing (Basoglu et al., 2005; Isogai et al., 2017). AMP readily forms adenosine *in situ* and thus most of its actions occur through ARs, but not all of its *in vivo* effects arise from AR activation (Carlin et al., 2018; Xiao et al., 2019).

##### Cancer

Adenosine 5′-triphosphate plays a physiological role in obstructive airway disease to increase inflammation and to induce bronchoconstriction and cough (Pelleg et al., 2016). ATP has been administered in humans intravenously in clinical trials for the treatment of both cachexia in cancer and the cancer itself (Rapaport et al., 2015). The working hypothesis was that by increasing the intracellular ATP pools in erythrocytes, the energy balance could be restored. Alternatively, an action on P2 receptors was considered, but the extracellular ATP pools were elevated only briefly in patients with advanced solid tumors. Nevertheless, with the ubiquitous presence of ectonucleotidases, one main action of ATP would be through increased adenosine acting at ARs.

#### Metrifudil **2**

##### Glomerulonephritis

Many adenosine derivatives found in early studies (1960s and 1970s) to be biologically active at what were later termed the A_1_AR and A_2A_AR, contain bulky, hydrophobic substitution at the adenine *N*^6^ position (Olsson, 2003; Jacobson and Gao, 2006; Mantell et al., 2010). This describes two potent agonists, Metrifudil **2** and *R*-PIA (*R*-*N*^6^-phenylisopropyladenosine (structure not shown), which are moderately A_1_AR-selective. Metrifudil (60 mg oral dose) was one of the earliest synthetic adenosine agonists to be studied in humans (Schaumann and Kutscha, 1972; Wilbrandt et al., 1972), along with *R*-PIA (Schaumann et al., 1972). Following the discovery that vasodilator dipyridamole (equilibrative adenosine transporter ENT1 inhibitor) was active against glomerulonephritis by an adenosinergic mechanism, Metrifudil was applied to its treatment, which resulted in limited improvement in three patients. However, no subsequent trials of Metrifudil or *R*-PIA were reported, probably because of their cardiovascular side effects.

### A_1_AR-Selective Agonists

#### Trabodenoson (INO-8875, PJ-875) **4**

##### Glaucoma

A_1_AR partial agonist Trabodenoson (INO-8875) **4**, which is a 5′-nitrate ester derived from CPA **3**, was in advanced clinical trials as an ophthalmic formulation for primary open-angle glaucoma and ocular hypertension. However, the development was terminated in 2017 following a Phase 3 trial due to failure to achieve its primary endpoint (Jacobson and Civan, 2016). Trabodenoson and its congeners were also considered for treatment of arrhythmias (Elzein and Zablocki, 2008; see below). Another analog containing a 5′-nitrate ester, but with a *N*^6^-3-aminotetrahydrofuryl group was found to lose its nitro group *in vivo* to form the parent full agonist, which was associated with side effects.

#### CHA **5**

##### Hypothermia

The use of A_1_AR agonist CHA **5** for inducing therapeutic hypothermia has been proposed (Jinka et al., 2015). However, it is likely that the hypothermic effect of CHA in rodents may be through both a peripheral A_3_AR, and a central A_1_AR (Carlin et al., 2017).

#### SDZ WAG994 **6**

##### Diabetes and obesity

A_1_AR is highly expressed in adipose tissue and involved in triglyceride (TG) storage. Breakdown of TG and the subsequent increase in plasma free fatty acids (FFA) results in development of insulin resistance in peripheral organs. Insulin resistance is associated with obesity and development of type 2 diabetes (Antonioli et al., 2015). Adenosine-mediated A_1_AR activation or A_1_AR overexpression in adipocytes results in suppression of lipolysis and reduction of plasma FFA. Various adenosine analogs have been developed for their potential intervention in diabetes. *N*^6^-Cyclopentyladenosine (CPA, **3**) reduced FFA and cholesterol levels and increased glycogen synthesis in skeletal muscle in streptozotocin (STZ) diabetic rats (Cheng et al., 2000). Thus, A_1_AR agonists may have beneficial effects on glucose utilization by peripheral tissues to lower plasma glucose.

Typically, both 2′- and 3′-hydroxyl groups are important for nucleoside recognition in the AR binding sites. SDZ WAG994 **6** is a structurally unusual 2′-*O*-methyl A_1_AR agonist that did not display hemodynamic side effects in humans. However, SDZ WAG994 increased PR interval, consistent with A_1_AR activation in the AV node and its suggested use in tachycardia (Jacobson and Knutsen, 2001).

SDZ WAG994 **6** was also under development as an antilipolytic agent for treating diabetes (Ishikawa et al., 1998). Oral administration of SDZ WAG994 and another A_1_AR agonist Selodenoson (RG14202, **14**) in STX-treated rats decreased FFA, TG levels and heart rate in a dose-dependent manner (Cox et al., 1997). A human study revealed that RPR749 (structure not shown), was capable of decreasing circulating FFA levels and thus may be beneficial in treating hyperlipidemia (Shah et al., 2004). These compounds have therapeutic potential for the treatment of cardiovascular and metabolic disorders.

#### Cl-ENBA **7** and MRS7469 **8**

##### Hypothermia and pain

Several A_1_AR agonists, Cl-ENBA **7** and MRS7469 **8**, were recently reported to activate in the mouse A_1_AR in the brain when administered peripherally, without comparable peripheral A_3_AR activation (Carlin et al., 2017; Tosh et al., 2019). Cl-ENBA **7** is a highly A_1_AR-selective adenosine agonist that is used as a pharmacological probe (Tosh et al., 2019), and its efficacy in pain models demonstrated (Luongo et al., 2012). It consists of a mixture of two diastereoisomers, and thus its *in vivo* A_1_AR target engagement is complicated. However, when administered intraperitoneally in mice, it activated the central A_1_AR preferentially over the peripheral A_3_AR in mast cells, due partly its being a full agonist for the A_1_AR but a partial agonist of low efficacy or an antagonist at the A_3_AR (Carlin et al., 2017). This is consistent with a report that *S*-*N*^6^-endo-norbornyl-adenosine (*S*-ENBA) is a full agonist at the A_1_AR but low-efficacy partial agonist at the A_3_AR (Gao et al., 2003).

Like Cl-ENBA, MRS7469 **8** is a highly selective agonist that activated the central A_1_AR preferentially when administered peripherally, leading to A_1_AR-dependent hypothermia and locomotor depression (Tosh et al., 2019). When administered icv. (52 μg/kg) it caused intense hypothermia. Thus, it crosses the blood brain barrier (BBB) sufficiently, given its high affinity (K_i_ 0.37 nM at mouse A_1_AR), to activate the A_1_AR. Moreover, MRS7469 with a non-chiral *N*^6^ group is a pure diastereoisomer, which is advantageous for *in vivo* studies.

#### GW493838 **9**

##### Pain

The A_1_AR is involved in depressant and protective functions in the brain and spinal cord, including suppressing pain (Sawynok, 1998; Zylka, 2011; Giorgi and Nieri, 2013). Intrathecal opioids induce local adenosine release (Eisenach et al., 2004), and exogenously applied adenosine and other A_1_AR agonists and PAMs reduce pain (Eisenach et al., 2013; Vincenzi et al., 2014). A_1_AR agonist GW493838 **9**, which is an adenosine analog highly modified at *N*^6^ and 5′ positions, showed efficacy in animal models of pain (Imlach et al., 2015). However, GW493838 (50 mg oral dose) failed to show significant efficacy in a clinical trial for treatment of chronic pain (diabetic pain). Also, a meta-analysis of patient postoperative pain in clinical data showed no analgesic effect of adenosine (Jin and Mi, 2017).

#### CVT-3619 (GS-9667) **10,** GR79236 **11** and ARA **12**

##### Diabetes

Increasing evidence indicates a crucial role of A_1_AR in the regulation of insulin sensitivity and glucose homeostasis especially in metabolically active organs such as adipose tissue, liver and skeletal muscle, which are related to diabetes mellitus (Peleli and Carlstrom, 2017). It has been convincingly demonstrated that A_1_AR is critical for regulation of lipid metabolism, and thus A_1_AR agonists have been proposed for the treatment of type II diabetes (T2D) and obesity (Antonioli et al., 2018). The white adipocyte A_1_AR inhibits lipolysis. Curiously, a functional A_2A_AR activates thermogenic brown adipose tissue (BAT) as indicated using human PET imaging (Lahesmaa et al., 2018), and A_2A_AR agonists might prove beneficial in metabolic conditions (Tozzi and Novak, 2017). Cold exposure in human subjects reduced PET ligand binding in BAT, indicative of elevated local adenosine release.

Several A_1_AR agonists, GR79236 **11**, ARA **12**, and CVT-3619 **10**, have been in clinical trials for T2D due to their ability to increase insulin sensitivity (Bigot et al., 2004; Kiesman et al., 2009; Staehr et al., 2013). However, development of full agonists, such as GR79236 and ARA, was not successful due to cardiovascular side effects (Elzein and Zablocki, 2008). Although both full and partial agonists may lower non-esterified fatty acid levels, it is suggested that partial agonists may improve insulin sensitivity without producing severe cardiovascular side effects (Elzein and Zablocki, 2008). A single dose of ARA given to healthy individuals during a phase I clinical trial reduced plasma FFA levels, but individuals developed tolerance to the drug (Zannikos et al., 2001). The A_1_AR partial agonist CVT-3619 (GS-9667) has been reported to lower FFA in both healthy and obese subjects without showing evidence of A_1_AR desensitization (Staehr et al., 2013), but oral doses of ≥ 300 mg were required to see the FFA effect. The individual benefits of GS-9667 and sitagliptin [Januvia, an inhibitor of DPP4 (dipeptidyl peptidase 4)] on glucose and lipid homeostasis were enhanced in combination (Ning et al., 2011).

#### Tecadenoson **13** and Selodenoson **14**

##### Arrhythmias

Despite its demonstrated A_1_AR-dependent beneficial effect in PSVT, adenosine is known to cause atrial fibrillation (AF) in about 15% of patients by decreasing the refractory period of the atrium and causes other adverse effects related to the activation of other AR subtypes (Glatter et al., 1999). Thus, extensive efforts have been made in developing selective A_1_AR agonists as anti-arrhythmic agents (Mason and DiMarco, 2009). A_1_AR full agonists Tecadenoson **13** (Corino et al., 2015), Selodenoson **14** and Trabodenoson **4** (Kiesman et al., 2009; Mason and DiMarco, 2009) have been under development. However, full agonists are known to cause tachyphylaxis, presumably due to A_1_AR desensitization. Tecadenoson has been in a Phase 3 trial for the termination of supraventricular tachycardia (SVT) (Elzein and Zablocki, 2008; Mason and DiMarco, 2009), but its development was discontinued in 2009. A clinical safety study of Tecadenoson for the treatment of AF was performed, but its clinical development was also curtailed.

#### NNC-21-0136 **15**

##### Stroke

NNC-21-0136 **15** is an A_1_AR selective agonist that was designed for neuroprotection. Its hemodynamic effects were minimal, revealing a brain-protective effect in stroke models (Knutsen et al., 1999; Jacobson and Knutsen, 2001), but it did not enter human testing.

#### MRS5474 **16**

##### Seizures and depression

A_1_AR agonists are of interest in the CNS for their anxiolytic, antinociceptive, antidepressant and antiseizure properties, and behavioral results with A_1_AR KO mice support the use of A_1_AR in this context. Unfortunately, many adenosine derivatives display minimal ability to cross the BBB (Schaddelee et al., 2005; Tosh et al., 2019). 4′-Truncated nucleosides, thionucleosides and methanocarba-nucleosides (containing a [3.1.0]bicyclohexyl ring system) were originally characterized as A_3_AR low-efficacy, selective partial agonists. However, an *N*^6^-dicyclopropylmethyl group present in MRS5474 **16** substantially shifts the selectivity toward A_1_AR, especially in mouse (204-fold compared to mouse A_3_AR), compared to other a-branched *N*^6^ groups (Tosh et al., 2012, 2019; Carlin et al., 2017). The small molecular weight (376), polar surface area (93 Å^2^) and number of H-bond donor groups (3) positioned MRS5474 for potential brain application. MRS5474 showed antidepressant activity in a mouse model that was mediated by homer1 protein in the medial prefrontal cortex, and upregulation of homer1 by an AR agonist was lost in A_1_AR KO mice (Serchov et al., 2015). Nevertheless, it also activated a peripheral mA_3_AR (Carlin et al., 2017).

#### Capadenoson **17** and Neladenoson **18**

##### Angina

Anti-ischemic effect of A_1_AR agonists has been demonstrated in animal studies, but clinical successes are lacking, and more relevant clinical models are needed (Borea et al., 2016; Lasley, 2018).

Most known AR agonists are adenosine derivatives, but two classes of pyridine-derived agonists are known (Guo et al., 2018). The non-nucleoside A_1_AR agonist Capadenoson **17** (BAY68-4986), having an atypical 3,5-dicyanopyridine structure, was evaluated in patients with stable angina using an oral dose of 4 mg, once daily (Kiesman et al., 2009; Tendera et al., 2012). However, Capadenoson was withdrawn from clinical trials for angina and for AF.

##### Heart failure

Rather than full agonists, an A_1_AR partial agonist Neladenoson **18** (in the form of a dipeptide ester prodrug **19**) is now being tested in patients with chronic heart failure (Greene et al., 2016; Dinh et al., 2017; Meibom et al., 2017; Voors et al., 2017). Compared with Capadenoson (Baltos et al., 2017), Neladenoson is a more selective partial agonist for A_1_AR. Neladenoson has been shown to improve cardiac function without producing bradycardia, atrioventricular blocks, or undesirable effect on blood pressure (Meibom et al., 2017; Voors et al., 2017). The rationale for using partial A_1_AR agonists is based on the observation that the activation of myocardial A_1_ARs by partial agonists protects cardiac function related to ischemia and reperfusion injury without producing severe side effects (Albrecht-Küpper et al., 2012; Voors et al., 2017). A multiple dose study of Neladenoson (BAY 1067197) (ParSiFAL, 5 – 40 mg oral dose, once daily) in heart failure is ongoing.

#### MMPD **20**

##### Imaging

Numerous A_1_AR ligands have been in development for potential use in diagnosis of various conditions, such as depression, Parkinson’s disease, Alzheimer’s disease, epilepsy, ischemia, and sleep disorders. The A_1_AR is highly expressed in many brain regions, such as the hippocampus, neocortex, thalamus and basal ganglia (Fredholm et al., 2001). *In vivo* imaging of A_1_AR in the human brain is therefore an attractive approach for diagnosis, and various AR agonists and antagonists have been developed for PET brain imaging (van Waarde et al., 2018). Although varied AR subtype selectivities and agonist efficacies are seen with the class of atypical 3,5-dicyanopyridine ligands, partial agonist MMPD **20** was recently shown to be highly A_1_AR selective, with 16% maximal human A_1_AR activation, and suitable for the PET imaging in the rat brain (Guo et al., 2018). It has a relatively low molecular weight (373) and polar surface area (108 Å^2^), which allows it to cross the BBB. Typical ribose-containing A_1_AR agonists have limited utility to be administered therapeutically for CNS treatment due to their low degree of brain uptake from the periphery (Schaddelee et al., 2005).

### A_2A_AR-Selective Agonists

#### Regadenoson **21**

##### Imaging

Regadenoson **21** (CVT-3146, Lexiscan) is a moderately selective, short acting A_2A_AR agonist that is administered i.v. for MPI (Palani and Ananthasubramaniam, 2013). It was first approved as a pharmacologic stress agent in 2008. At present, it is the only synthetic AR agonist that is approved for human use, although it is not highly potent or selective for the A_2A_AR. Nevertheless, Regadenoson’s A_2A_AR selectivity is higher in human than in mouse, in which it is actually 10-fold A_1_AR selective compared to A_2A_AR (Carlin et al., 2018). The availability of an FDA-approved new chemical entity (NCE) allows it to be tested in diverse clinical trials for cardiovascular treatment and diagnosis (>60 currently listed in ClinicalTrials.gov, accessed 12-31-2018).

##### Sickle cell disease

In addition to their diagnostic application in MPI, A_2A_AR agonists have been considered for treatment of inflammation (Cekic and Linden, 2016) and sickle cell disease (SCD, Field et al., 2014). A_2A_AR activation in natural killer T (iNKT) cells is anti-inflammatory as demonstrated in a transgenic mouse model of SCD. However, A_2B_AR activation in erythrocytes is predicted to have a harmful effect in SCD. The effects of Regadenoson **21** as an A_2A_AR agonist in SCD patients were evaluated in a clinical trial, but there was no statistically significant benefit (Field et al., 2017).

##### Lung transplantation

Murine lung ischemia reperfusion injury occurring via NADPH oxidase 2 (NOX2) and IL-17 is also attenuated by A_2A_AR agonist **26** (Sharma et al., 2016), which has led to an ongoing clinical trial of Regadenoson **21** in lung transplantation. The safety of using Regadenoson for MPI in patients with mild to moderate COPD and asthma was established (Golzar and Doukky, 2014).

##### Glioblastoma

A_2A_AR agonists transiently increase BBB permeability (Kim and Bynoe, 2016), and this is being evaluated as a novel pharmacological approach to drug delivery to the brain. Regadenoson was tested clinically in an attempt to raise the concentration of the anticancer drug temozolomide in the brain interstitium, determined using microdialysis in glioblastoma patients (Jackson et al., 2018).

#### Spongosine (BVT.115959, CBT-1008) **22** and Other Naturally Occurring AR Agonists

##### Pain

Numerous other A_2A_AR agonists were studied in preclinical testing or clinical trials prior to the approval of Regadenoson. Among the first such agonists was the simple 2-methoxy derivative of adenosine, spongosine (BVT.115959) **22**, a marine natural product (García et al., 2018). Actually, spongosine is slightly selective for and equipotent at the human A_1_AR and A_3_AR. It was shown to be effective in a clinical trial for diabetic neuropathic pain (7 mg oral dose, 3X daily), which was terminated because the company discontinued small molecule research (Knezevic et al., 2015).

##### Inflammation

Activation of the A_2A_AR by endogenous adenosine provides benefit in animal models of inflammation and rheumatic disease, for example in rat models of osteoarthritis (Cronstein and Sitkovsky, 2017; Haskó et al., 2018). Other naturally occurring adenosine or deoxyadenosine derivatives have been applied as AR agonists. Polydeoxyribonucleotide (PDRN, structure not shown), of molecular weight 80–200 KD and extracted from trout or salmon sperm, is degraded by plasma DNA nucleases or cell membrane-bound nucleases giving rise to nucleosides and nucleotides. It is asserted that the degraded products pharmacologically activate the A_2A_AR, based on antagonism by the relatively weak and non-selective A_2A_AR antagonist 3,7-dimethyl-1-propargylxanthine (DMPX). PRDN’s therapeutic effects include tissue repairing, anti-ischemic, and anti-inflammatory, making it suitable in regenerative medicine and for treating diabetic foot ulcers. Topically applied PDRN was in a clinical trial for reducing inflammation to promote wound healing in cases of diabetic foot ulcers (Squadrito et al., 2017). Also, topically applied PDRN significantly reduced pain and increased joint function in an animal model of osteoarthritis and increased neurogenesis in a spinal cord injury model (Irrera et al., 2018).

#### Sonedenoson (MRE-0094) **23** and Binodenoson (WRC-0470, MRE-0470) **24**

##### Imaging

Many adenosine derivatives that proved to be A_2A_AR-selective agonists have bulky, hydrophobic substitution at the C2 position of adenine (Jacobson and Gao, 2006). A_2A_AR agonist Binodenoson **24** (≤ 1.5 μg/kg, i.v.) administered for MPI of patients with coronary artery disease did not cause the side effect of bronchoconstriction (Murray et al., 2009).

##### Wound healing

Sonedenoson (MRE-0094) **23** was effective in the treatment of poorly healing wounds in animal models (Victor-Vega et al., 2002), an A_2A_AR-agonist effect later found to be dependent on tissue plasminogen activator (Montesinos et al., 2015). A Phase 2 clinical trial of Sonedenoson administered as a topical gel for diabetic foot ulcers had poor enrollment and was terminated in 2008.

#### Apadenoson (ATL-146e, BMS 068645) **25** and Evodenoson (ATL-313, DE-112) **26**

##### Imaging

Apadenoson **25** (Rieger et al., 2001; Zoghbi and Iskandrian, 2012) was in several clinical trials for MPI and SCD, which has a component of hypoxia. Apadenoson contains a labile ester moiety, which is cleaved *in vivo* to limit its duration of action. Its more stable, urethane-containing congener Evodenoson (ATL-313) **26**, was developed as a candidate drug for treating multiple myeloma (Rickles et al., 2010; van Waarde et al., 2018).

#### UK-371104 **27** and UK-432097 **28**

##### Pulmonary inflammation

Intratracheal administration of A_2A_AR agonist UK-371104 **27**, with sterically bulky *N*^6^ and C2 substituents, in anesthetized guinea pig, inhibited the capsaicin-induced bronchoconstriction without affecting blood pressure (Trevethick et al., 2008). Thus, additional lung-focused A_2A_AR agonists were explored for treating lung inflammation.

UK-432097 **28** is a selective A_2A_AR agonist that was in a failed clinical trial for COPD (Mantell et al., 2010), although its pharmacology is comparable to its preceding congener, UK-371104 **27**. UK-432097 as an inhaled dry powder was not efficacious in human trials (discontinued in 2008), possibly due to its agonist activity at the A_1_ and A_3_ARs, and/or its high MW (778), and multiple H-bond donor (7) and acceptor (13) groups reduced its bioavailability, even when administered directly in the lungs by inhalation. However, it displays a favorably slow off-rate from the receptor, which has been suggested to contribute to its sustained agonist effects (Hothersall et al., 2017). The extended *N*^6^ and C2 substituents of UK-432097 and its congeners interact with A_2A_AR extracellular regions to impede their dissociation. The bulky *N*^6^ and C2 substitutions of UK-432097 enabled the structural determination of its A_2A_AR complex (Xu et al., 2011).

#### GW328267X **29**

##### Asthma and allergy

A dual A_2A_AR agonist and A_3_AR antagonist GW328267X **29** failed to show efficacy in a clinical trial for asthma (inhaled) and allergic rhinitis (intranasal) (Trevethick et al., 2008), despite its anti-inflammatory efficacy in animal models. Its structure is unusual in that it contains an ethyl-tetrazole group at the ribose 4′ position, thus contributing to its dual action at the two AR subtypes (Trevethick et al., 2008). Its side effects (hypotension, tachycardia) even when administered by inhalation were dose-limiting in the clinical trials. However, intravenous infusion of GW328267X in humans (52 μg/kg, over 5.5 h) resulted in a mechanism-related tachycardia that was ascribed to A_2A_AR activation in the carotid bodies, which was not alleviated upon prolonged agonist exposure. The lack of tachyphylaxis leading to prolonged tachycardia was not acceptable (Allen et al., 2013), and its clinical testing was discontinued. The translational failure of A_2A_AR agonists is likely due to their limited selectivity, especially their agonist activity at the A_1_AR. It has been suggested that A_1_AR antagonists and A_2A_AR agonists may have beneficial effects for asthma (Gao and Jacobson, 2017).

### A_2B_AR-Selective Agonist

#### BAY 60-6583 **30**

##### Ischemia, inflammation, diabetes, asthma, and cancer

Although there are no A_2B_AR agonists currently in clinical evaluation, animal models suggest its activation might result in beneficial effects in acute lung injury, ischemia and vascular leakage (Eltzschig et al., 2003; Eltzschig, 2009). The non-nucleoside (3,5-dicyanopyridine) agonist BAY 60-6583 **30** has been used as an *in vitro* and *in vivo* pharmacological probe, although its degree of efficacy and its species dependence of affinity/selectivity can vary (Gao et al., 2014). In some models, including insulin release in MIN6 mouse insulinoma cells, the compound was reported to act as an A_2B_AR antagonist. Therefore, highly selective and reliably efficacious A_2B_AR agonists are still lacking. Moreover, the signaling pathways activated or inhibited by the nominally G_s_-coupled A_2B_AR are complex and involve multiple G proteins (Gao et al., 2018).

Mast cell A_2B_AR activation might be useful in the treatment of asthma (Gao and Jacobson, 2017). In the intestines, kidney and other organs, this receptor has an anti-ischemic effect (Grenz et al., 2008; Hart et al., 2011). A_2B_AR activation is predicted to have beneficial cardiovascular effects and maintain the endothelial cell barrier (Eltzschig et al., 2003). BAY 60-6583 was shown to have protective effects in a model of myocardial reperfusion injury (Tian et al., 2015). A_2B_AR activation leading to the PI3K/Akt pathway is anti-inflammatory by shifting macrophages to an M2 phenotype. Pre-ischemic administration of BAY 60-6583 stimulated leukocyte PI3K/Akt in the mouse spleen to reduce myocardial reperfusion injury in an IL-10-dependent manner (Ni et al., 2018).

A_2B_AR activation might also be useful in treating T2D and atherosclerosis, and preventing vascular lesions due to smooth muscle cell proliferation after angioplasty (Koupenova et al., 2012; Sun and Huang, 2016). A_2B_AR KO mice displayed increased fatty liver pathology, tissue inflammation and insulin resistance due to the lack of this receptor in macrophages (Johnston-Cox et al., 2014). A_2B_AR activation reduced inflammation and macrophage activation resulting from FFA (Csóka et al., 2014). The receptor was highly upregulated in mice subjected to a high-fat diet (HFD), and A_2B_AR KO mice on this diet developed obesity and insulin resistance. BAY 60-6583 administered for 4 weeks HFD restored endocrine function and reduced inflammation. However, A_2B_AR gene expression was found to be elevated in cases of human gestational diabetes, but this observation did not establish whether an A_2B_AR agonist or antagonist would be more beneficial (Wojcik et al., 2014).

Although blocking the A_2B_AR is considered a target in conjunction with cancer immunotherapy, its activation also has been reported to reduce proliferation of cancer cells (Koussémou et al., 2018). A_2B_AR activation led to ERK1/2 dephosphorylation and reduced cell proliferation through inhibition of the MAPK signaling pathway in the MDA-MB-231 breast cancer cell line.

### A_3_AR-Selective Agonists

#### IB-MECA **31**

##### Autoimmune inflammatory diseases

A_3_AR agonists display anti-inflammatory and anticancer effects in various *in vivo* disease models (Cronstein and Sitkovsky, 2017; Jacobson et al., 2018). A_3_AR agonists stimulate chemotaxis in neutrophils through the leading edge, which could be pro-inflammatory. However, systemic A_3_AR agonist administration could actually have an anti-inflammatory effect by inhibiting neutrophil chemotaxis because of the non-directional agonist exposure (Chen et al., 2006).

IB-MECA (CF101, Piclodenoson) **31**, the first moderately selective A_3_ agonist (Gallo-Rodriguez et al., 1994), is being developed for the treatment of autoimmune anti-inflammatory diseases, including rheumatoid arthritis (RA) and psoriasis (both in Phase 3) (Fishman et al., 2012). In Phase 2 trials, its action in RA and psoriasis compared favorably to existing treatments for those conditions, but it did not display serious adverse effects, as do the current treatments. In a comparison of 1, 2, and 4 mg oral IB-MECA doses in a 12-week Phase 2 psoriasis trial, the greatest patient improvement was observed with the 2 mg dose (Fishman et al., 2012). Similarly in a Phase 2 RA trial, the middle (1 mg, compared to 0.1 and 4 mg) oral dose achieved the highest responses. Peripheral blood mononuclear cells (PBMCs) from psoriasis patients showed elevated A_3_AR expression. IB-MECA inhibited proliferation and formation of IL-17 and IL-23 in a human keratinocyte cell line (Cohen et al., 2018). IB-MECA was previously in Phase 2 clinical trials for dry eye disease and glaucoma, 1 mg and 2 mg, respectively (oral, twice daily), which failed to demonstrate efficacy (Avni et al., 2010; Jacobson and Civan, 2016).

#### Cl-IB-MECA **32**

##### Liver diseases

Cl-IB-MECA **32**, was initially shown to display a higher A_3_AR agonist selectivity than IB-MECA at the rat ARs. However, at the mA_3_AR, IB-MECA is more potent and selective than Cl-IB-MECA (Carlin et al., 2017). Cl-IB-MECA (CF102, Namodenoson) is being developed for the treatment of liver conditions, including hepatocellular carcinoma (HCC) and non-alcoholic steatohepatitis (NASH) (Fishman et al., 2018). A_3_AR agonists have apoptotic and anticancer effects *in vivo* induced by Wnt signaling deregulation (Bar-Yehuda et al., 2008). The US Food and Drug Administration and the European Medicines Agency granted fast track designation to **32** for the treatment of liver cancer. Cl-IB-MECA (up to a 25 mg oral dose) increased the median overall survival in patients with advanced HCC by 7.8 months in patients (Stemmer et al., 2013), which was improved over the current treatment. There were no serious adverse effects or dose-limiting toxicity. Secondarily, the trial examined using the A_3_AR as a predictive marker of the CF102 clinical response. The use of A_3_AR to prevent cytokine release syndrome in cancer immunotherapy has been proposed (Cohen and Fishman, 2019).

An anti-steatotic effect of Cl-IB-MECA in an HFD mouse model of NASH, induced by STZ administered 2 days after birth, was mediated via a molecular mechanism leading to decreased α-smooth muscle actin (αSMA, a marker of pathological fibroblasts) and cytokeratin 18 (CK-18, a predictor of NASH severity). A Phase 2 trial of CF-102 (12.5 and 25 mg oral doses, twice daily) for NASH treatment is underway.

##### Skeletal muscle protection

A_3_AR agonists, including Cl-IB-MECA, protect skeletal muscle in ischemic models in a phospholipase C-b2/b3-dependent manner (Zheng et al., 2007).

#### CP-608,039 **34** and CP-608,039 **35**

##### Cardioprotection

DeNinno et al. (2003) reported that CP-608,039 **35** is a highly A_3_AR selective and water-soluble agonist that was being evaluated for the prevention of perioperative myocardial ischemic injury. This follows numerous other reports showing that A_3_AR activation protects ischemic cardiomyocytes by preconditioning (Lasley, 2018).

Recently, selective A_3_AR deletion in mouse cardiomyocytes was used to demonstrate that activation by selective agonist CP-532,903 (**34**) of a myocardial A_3_AR provides ischemic tolerance that is dependent on K_ATP_ channels (Wan et al., 2019). This study resolves a long-standing controversy by showing that a protective A_3_AR is present in adult ventricular cardiomyocytes, although expressed at very low levels (copy number of 85 per 100 ng total RNA versus 12,830 for the A_1_AR).

#### MRS5698 **36**

##### Pain

As noted above, A_1_AR agonists and PAMs are already under consideration for pain treatment. The efficacy of A_3_AR agonists for chronic pain was first explored depth in 2011 (Chen et al., 2012). The approach of using A_3_AR agonists for pain treatment was initially controversial, as A_3_AR activation had been described in earlier review papers as an uninteresting target or even an anti-target for pain relief (Nascimento et al., 2012; Janes et al., 2016). Reasons for this premature characterization were: truly selective A_3_AR agonists were not initially available and the A_3_AR causes release of inflammatory mediators, e.g., histamine and serotonin, from peripheral mast cells in rodents, but not human and other species (Auchampach et al., 1997; Leung et al., 2014; Gao and Jacobson, 2017). These mediators can contribute to inflammation in mouse and rat, and Sawynok (1998) concluded that A_3_AR activation induces pain and paw oedema. However, consistent with the now well-documented action of A_3_AR activation in various chronic pain models, A_3_AR KO mice had a lower pain threshold in the hind-paw hot-plate test (40% greater latency, Fedorova et al., 2003). Curiously, there was no difference between A_3_AR KO and WT mice in the acute pain response in the tail-flick test.

The two selective A_3_AR agonists in already clinical trials, IB-MECA and Cl-IB-MECA, at high doses *in vivo* might interact with other ARs, as has been observed in experimental models (Fozard, 2010). A new series of C2-extended (N)-methanocarba analogs displayed even greater A_3_AR selectivity, estimated to be in the range of at least 10,000-fold in comparison to other ARs (Jacobson et al., 2018). Among these agonists is MRS5698 **33**, which has been shown to reduce chronic neuropathic pain, including oxaliplatin-induced neuropathic pain (Little et al., 2015; Wahlman et al., 2018). MRS5698 has many drug-like properties – it is non-toxic and relatively stable *in vivo*, except that its oral bioavailability in the rat is only 5%F (Tosh et al., 2015). Nevertheless, by various modes of administration it is efficacious in pain models *in vivo*, including chronic constriction injury-induced, chemotherapy-induced and cancer-induced. Its ability to reduce chronic hyperalgesia is not affected by the A_3_AR-induced histamine release observed in rodent but not human mast cells (Carlin et al., 2016).

#### MRS5980 **37**

##### Pain

MRS5980 **37** is a highly selective C2-arylethynyl (N)-methanocarba A_3_AR agonist, which has been demonstrated to be highly efficacious in *in vivo* pain models following administered by oral gavage, with a protective effect lasting up to 3 h (Tosh et al., 2014; Janes et al., 2016), and its metabolomics has been studied (Fang et al., 2015). The 2-chlorothienyl group is stable *in vivo*, and the aryl alkyne group was shown to be not highly reactive. MRS5980 and other A_3_AR agonist were shown to indirectly block a pro-nociceptive N-type Ca^2+^ calcium channels, a proven target in controlling pain, and cell excitability in the spinal cord dorsal horn (Coppi et al., 2019). Thus, therapeutic application of A_3_AR agonists appears to be a promising approach for treating pain of different etiologies.

#### LJ-529 **33** and MRS4322 **38**

##### Stroke

von Lubitz et al. (1999) found that both A_1_AR and A_3_AR agonists have cerebroprotective properties in a model of gerbil forebrain ischemia. An (N)-methanocarba nucleoside MRS4322 **38** was proposed in a patent application (Korinek et al., 2018) as a treatment for stroke and traumatic brain injury. The nucleoside appears to act through the A_3_AR, to which it binds in the μM range, because a selective A_3_AR antagonist propyl 6-ethyl-5-((ethylthio)carbonyl)-2-phenyl-4-propylnicotinate (MRS1523) diminished the benefit of reduced stroke lesions. LJ-529 **33** (4′-thio-Cl-IB-MECA) is a selective A_3_ adenosine agonist that was shown to be protective in a rat stroke model and inhibited brain migration of inflammatory cells (Choi et al., 2011). However, platelet A_2A_AR activation by LJ-529 increased the bleeding risk.

### AR Allosteric Enhancers (PAMs)

**FIGURE 3:**
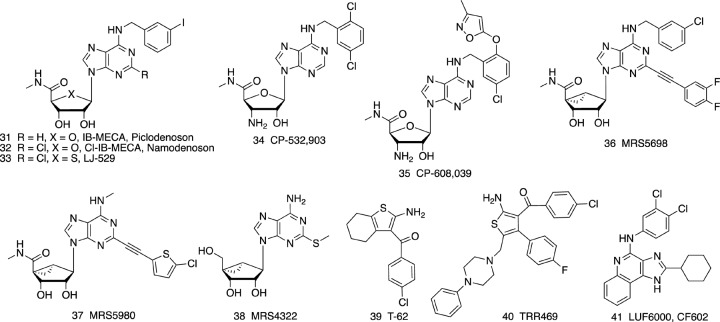
A_3_AR- (**31**-**38**) selective agonists and allosteric enhancers (PAMs) of the A_1_AR (**39**, **40**) and A_3_AR (**41**).

PAMs of the A_1_AR and A_3_AR have been the subject of preclinical and clinical evaluation (Figure 3 and Table 2). PAMs, in principle, may remain silent until a large rise in the extracellular adenosine occurs, at which time the PAM would amplify adenosine’s action at a particular AR subtype. Thus, PAMs are described as temporally and spatially specific modulators (Gao et al., 2011). Also, PAMs tend to be more subtype selective than orthosteric agonists, because their non-canonical binding regions on the GPCRs are those that have the most diverse sequences within the receptor family (Vecchio et al., 2018).

#### T-62 **39**

Benzoylthiophenes are the earliest and most extensively studied class of A_1_AR PAMs, and they have allosteric agonist properties as well as enhancing the effect of other A_1_AR agonists (Vincenzi et al., 2014; Jacobson and Gao, 2017). The benzoylthiophenes Just as A_1_AR agonists have been considered for pain treatment, a representative benzoylthiophene T-62 **39** entered a clinical trial for postherpetic neuropathic pain in 2008 that was discontinued because of its lack of efficacy (Giorgi and Nieri, 2013). Also, some patients displayed transient, elevated liver transaminases. Nevertheless, T-62 reduced hypersensitivity in animal models of neuropathic pain. The structural basis for recognition of benzoylthiophene PAMs involving A_1_AR extracellular loops (ELs) has been predicted using Gaussian accelerated molecular dynamics (Miao et al., 2018).

#### TRR469 **40**

##### Pain

A later generation benzoylthiophene TRR469 **40** was shown to be an A_1_AR PAM that increases the affinity of A_1_AR agonists (Vincenzi et al., 2014, 2016). It reduced pain, comparably to morphine, in writhing and formalin tests and in chronic STZ-induced diabetic neuropathy, and it displayed fewer behavioral side effects than an A_1_AR orthosteric agonist. Furthermore, the same A_1_AR PAM was anxiolytic in four behavioral models in the mouse, in a manner comparable to the anxiolytic drug diazepam (Vincenzi et al., 2016). This activity of **40**, which was blocked by a selective A_1_AR antagonist (DPCPX), is consistent with the previously noted anxiolytic activity of A_1_AR agonists.

#### LUF6000 **41**

##### Inflammation and erectile dysfunction

LUF6000 **41** is a imidazoquinolinamine A_3_AR allosteric enhancer (PAM). It enhanced the maximal efficacy of A_3_AR agonists but had no agonism on its own (Gao et al., 2011). Its interaction with the A_3_AR was species-dependent (Du et al., 2018). Although it was more efficacious in human, canine and rabbit than in rodent species, it was shown to produce an anti-inflammatory effect in rat models of adjuvant-induced arthritis and iodoacetate-induced osteoarthritis and in a mouse model of concanavalin A-induced liver inflammation (Cohen et al., 2014). The molecule is termed CF602 and is on a translational path for treatment of erectile dysfunction (Cohen et al., 2016).

## Conclusion

The structural and pharmacological features of key AR agonists and positive allosteric modulators (PAMs) have been summarized, with an emphasis on molecules that have been in humans or that were considered for human testing. From the thousands of selective AR agonists or allosteric enhancers reported, there are few translational successes. Many AR agonists have been in clinical trials for disease treatment or diagnosis, but only two are approved for human use, i.e., short-acting agonists adenosine and Regadenoson. However, new concepts and compounds are currently being developed and applied toward preclinical and clinical evaluation, and initial results are encouraging. AR agonists for treating inflammation, pain, cancer, NASH, angina, sickle cell disease, ischemic conditions and diabetes are under development. Multiple clinical trials with two A_3_AR agonists are ongoing.

## Author Contributions

KJ organized the outline and wrote most of the text. DT contributed to the writing and researching the topic. SJ contributed to the writing and researching the topic. Z-GG contributed to the writing and researching the topic.

## Conflict of Interest Statement

The authors declare that the research was conducted in the absence of any commercial or financial relationships that could be construed as a potential conflict of interest.
